# The social origins of consciousness

**DOI:** 10.1098/rstb.2024.0300

**Published:** 2025-11-13

**Authors:** Kristin Andrews, Noam Miller

**Affiliations:** ^1^ Department of Philosophy, York University, Toronto, Ontario M6H 3N8, Canada; ^2^ Graduate Center, New York., City University New York, Graduate Center, New York 10016, USA; ^3^ Department of Psychology, Wilfrid Laurier University, Waterloo, Ontario N2L 3C5, Canada

**Keywords:** consciousness, sentience, sociality, evolutionary function

## Abstract

We present the social origins of consciousness hypothesis, according to which the ability to coordinate with group members was the original adaptive function of consciousness. We offer three arguments. The phylogenetic argument presumes that consciousness is widespread among existing animals, and that widespread capacities are probably evolutionarily old. Early animals relied on consciousness to solve a problem that arose during the Cambrian, when animals first became behaviourally flexible—how to predict others’ behaviour and stay together as a group. The argument from neuroscience points to evidence that even very simple brains have the capacities for social rewards and pains, and that modern brains retain close connections between the substrates for social cognition and affect. The deep adaptive alignment between social pain and harm to animals develops an argument originally proposed by William James (1890). We provide evidence that in preference tests, bodily pain is preferred to social pain in a wide range of species. We offer two approaches to testing the hypothesis—the salience of social stimuli test and the overattribution of agency test. Working under the social origins of consciousness hypothesis could lead to significant breakthroughs in research, especially by focusing on simpler systems than are currently studied.

This article is part of the theme issue ‘Evolutionary functions of consciousness’.

## 1. Why is anything conscious?

Consciousness is identified first and foremost through first-person experience, not via some externally observable behaviour with a clear functional contribution. We feel its existence, but do not know what it is for. Despite this uncertainty, scientific and philosophical consciousness research always involves at least some implicit assumption about its function, and about the observable effects of conscious experience. These assumptions are part of the framework of consciousness research programmes.

A familiar presumptive function for consciousness is that it helps individuals avoid painful stimuli and seek out pleasurable ones. Such views tend to emphasize the pain caused by bodily damage, and the pleasures associated with bodily health. Consider William James’ adaptive alignment argument against consciousness epiphenomenalism. James observed that what the body needs tends to feel good, and what is harmful to the body tends to feel bad:

It is a well-known fact that pleasures are generally associated with beneficial, pains with detrimental, experiences … An animal that should take pleasure in a feeling of suffocation would, if that pleasure were efficacious enough to make him immerse his head in water, enjoy a longevity of four or five minutes … if pleasures and pain have no efficacy, one does not see … why the most noxious acts, such as burning, might not give thrills of delight, and the most necessary ones, such as breathing, cause agony. (James, 1890/1981 [1, p. 146])

This focus on bodily health and harms naturally leads to certain research programmes and experiments: for example, the current focus on pain in response to tissue damage, shock, or burning acid found in the animal literature reflects this presumption about the function of consciousness. However, this approach may be missing another more primitive function for consciousness, one that focuses on the psychological pains and pleasures associated with sociality.

Here, we propose the Social Origins of Consciousness hypothesis. Contemporary science offers us evidence that consciousness may exist widely across animal species [2], and philosophical argument supports the idea that all existing animals are conscious [3]. When a capacity is widely distributed, it is probably evolutionarily old. Thus, to ask whether there is some content that can be identified with the original function of consciousness, we turn to examine the point at which consciousness may have first emerged in early evolution. Our proposal is that when behavioural flexibility emerges we find the first instances of true sociality, and that consciousness serves the social function of identifying and responding to the behaviour of social partners. This point in evolutionary history is pivotal, because it is the instant at which simpler methods of coordinating behaviour no longer suffice.

We offer three arguments for the social origins of consciousness. In §2, we describe the hypothesis, and in §3, we present the first argument, where we develop a phylogenetic story of the origins of consciousness. We ground this phylogeny in known behavioural and anatomical traits of extant taxa, where possible, keeping in mind that such extrapolations can be tenuous. The story begins during the Cambrian explosion (CE) (~530 million years ago), but we first set the stage by describing pre-Cambrian conditions. In §4, we elaborate on this story, suggesting some of the ways in which early brains could have served as substrates for consciousness and examining whether remnants of this early structure can be found in contemporary brains. In §5, we build on James’ adaptive alignment argument, reviewing contemporary evidence that existing animals demonstrate a deep affective need for social partners, choosing social contact over basic physical needs. In §6, we propose how this hypothesis could be tested in a wide range of animal species.

Before we begin, a few preliminary remarks. First, we take consciousness to be an umbrella term that encompasses many dimensions, including sentience (feeling pain, pleasure, sensations), imaginings, dreamings, mental time-travel, self-awareness, inner speech and so forth. With the emergence of any one of these dimensions, a system becomes conscious. In what follows, we work from the premise that the earliest type of consciousness was sentience—the capacity for feeling negatively and positively valenced sensations, such as pain and pleasure. We anticipate that the different dimensions of consciousness will have their own adaptive functions, especially when it comes to inner speech or self-awareness, and that these emerged much later in evolutionary history and are not apparent in all extant species.

Second, our proposal does not address the function of these other dimensions and does not deny functional pluralism, but it is focused on the adaptive advantages associated with the emergence of sentience. This focus should not be taken to imply that sociality is the *only* function of consciousness experience. Consciousness today has a number of different adaptive functions in different domains, which is to be expected given that consciousness now has a large number of different dimensions [4,5]. This functional pluralism [6] offers consciousness researchers many paths for investigating consciousness in different domains such as visual processing, emotional expression, memory and social cognition. We also do not claim that sociality is necessary for consciousness, as the phylogenetic story of life on this planet may not be a universal one. Neither do we claim that an extant species that was truly asocial would lose consciousness, as consciousness would have been exapted to serve other functions after its appearance—again, today’s functional pluralism identifies many roles for consciousness to play.

Finally, we note that our proposal takes a very different approach from theories of consciousness that identify some particular neurophysiological structure or process as necessary for consciousness. We take all such accounts to be premature, given that we cannot know what features are necessary for consciousness before knowing which beings are conscious. A function first approach that is open to the possibility that the functional contributions of consciousness are multiply realizable offers a different path forward for the study of consciousness, and this is the path we take in this article.

## 2. The social origins of consciousness hypothesis

According to the social origins of consciousness hypothesis, the ability to coordinate with group members was the original adaptive benefit for consciousness. Organisms experienced negative feelings when distant from or out of sync with group members, modified their behaviour to regain proximity and experienced positive feelings when close to the group or coordinating with it. By taking the ability to predict and coordinate behaviour with other cognitive beings as a significant step in the evolution of consciousness, we offer a new lens through which to study consciousness in extant organisms.

We intend our story to be an invitation to think more broadly about consciousness as primarily a capacity of social systems [7]. This may seem somewhat paradoxical, since the largest barrier to the scientific study of consciousness comes from its nature as a subjective, first-person phenomenon, which is not currently measurable or observable—and hence not socially available. The subjective nature of consciousness may make individualistic approaches to the function of consciousness tempting, but we think this temptation must be resisted. Thus, our proposal is in tension with Cartesian views that take knowledge of the self to be primary, and with psychological views proposing that sophisticated cognitive capacities such as theory of mind or shared intentionality are needed for true sociality. That is, a familiar story is that the original function of consciousness was to support individual learning about environmental contingencies and that self-consciousness preceded an understanding of other agents as having motivations for their complex behaviours. The social origins of consciousness hypothesis flips this view, by proposing that consciousness first served to improve predictions about the behaviours of others, and that it was only later (possibly much later) that consciousness turned inwards towards the self. If anything, the hypothesis suggests that individual action and self-consciousness or separating oneself from the group, may have been the cognitive achievement.

This story relies on there being a major transition in the dynamics of group living with the emergence of cognitively flexible behaviour. This transition involves a separation between the processes of sensation and action, allowing for arbitrary intervals between the two. This gap is filled by the processes we call cognition. Before animals became cognitive beings, their motion was directly and immediately caused by external stimuli, without intervening representations. There was no learning, no memory and—we are hypothesizing—no consciousness (or at least no adaptive function for consciousness). With the emergence of cognition, individuals’ responses to stimuli become more variable and therefore harder for others to predict. In parallel, muscles evolved and replaced cilia as the primary effectors, greatly increasing the scale and speed of action, along with the range of actions possible, which also made prediction more difficult. The emergence of cognition and faster forms of movement thus created new problems for socially living organisms, making prior methods for coordinating and predicting others’ movements no longer effective. Coordinated behaviours that previously emerged reliably from the aggregative effects of simple slow movement were thrown into disarray by individual variability in history and response biases.

We describe this as a problem for the organisms given that individuals risked losing the benefits of group living, and none of their old solutions sufficed. Consciousness had the function of solving this new problem. However, for it to appear as a problem, the organisms themselves had to care about social partners before they could learn new ways of coordinating with them. Having feelings about one’s social situation led animals to focus their attention on social stimuli, and devote a large part of their cognition to social coordination.

## 3. Phylogenetic argument: sociality and the Cambrian explosion

The evolutionary history of the CE has been interpreted in many different ways, but the general story goes something like this. Along with many other physical and physiological innovations, muscles first emerged at this time, allowing for faster and more vigorous movement, including larger organisms [8,9]. However, muscle movements must be coordinated to move the whole organism. This, on many accounts, was the impetus for the evolution of modern neurons ([10]; although simpler forms predate the Cambrian [11]), which coordinated muscles across larger bodies [12]. Actions also need to be appropriate to incoming sensory information, so neurons also serve to integrate sensations and connect them to action. At about the same time, distal senses—such as image-forming eyes—emerged [13]. The combination of distal senses and faster movement enabled predation [14], which in turn led to the evolution of defensive mechanisms (shells and burrowing both emerge in the CE) as well as weapons (hard mouth parts appear in arthropods in the CE, as do the first grabbing appendages [15]). This started the arms race between prey and predators, which partly provided the selective pressure for animals to get larger and better at moving. There are more ways to be successful as a predator, leading to an explosive radiation in body forms and phyla [9]. It is widely agreed that a corresponding change in behaviour and cognition went along with these physiological developments. Importantly, for our story, the combination of faster movement at a larger scale with increased processing capacity gave rise to the first behaviourally flexible organisms, heralding an era of extreme unpredictability.

### (a) Step 1: Before there was consciousness, there was group living

Even before the start of the Cambrian era, 650 million years ago, a wide range of Ediacaran animals lived together in groups [16]. These animals, with their fractal structures and early experiments with muscles, were attached to the sea bed, as is apparent at sites such as Mistaken Point, Newfoundland, where volcanic ash preserved the ecosystem at a moment in time [17]. Thus, group living is ancient. These observations are supported by theoretical arguments that suggest group living was in place early in evolutionary history.

First, consider that today group living is ubiquitous, apparent in every taxon, including bacteria, which create public goods and form cooperative groups ([18]; even pre-Cambrian prokaryotes collectively formed stromatolites; [19]) single-celled eukaryotes (such as *Dictyostelium*, which displays altruism [20,21]), plants thatcommunicate chemically about environmental conditions [22] and a vast array of animal species [23,24]. Even animals deemed solitary are coming to be seen as having a non-gregarious form of sociality or being only opportunistically gregarious, such as the octopuses of Jervis Bay [25]. Many of these groups diverged long before the onset of the Cambrian era. When a phenotype is as widespread and conserved as this one, it is reasonable to hypothesize that it emerged early in evolutionary history.

Second, most early animals—such as Ediacarans—had limited (or no) mobility, entailing that individuals remained close to their point of origin, resulting in aggregations of closely related individuals and enhancing opportunities for indirect fitness benefits of cooperation, as observed in bacteria [26]. Animals that move passively on ocean currents might also be swept into aggregates, as occurs in extant cnidarians [27].

Finally, group living offers benefits to organisms, many of them via mechanisms that require only extremely simple forms of social interaction. For example, predator dilution effects require only that individuals remain close to each other [28], as do some public goods in bacteria [18]. Trilobites aggregated, possibly via shared attraction to chemical cues, aligning the same way relative to a current, by maintaining physical contact while moving [29], or perhaps similarly to how ants (their descendants) employ pheromone trails and tandem running [30]. Initial benefits of this sort served to keep animals close together, later allowing for the scaffolding of more complex social interactions and their attendant benefits (‘many-eyes’ effects, collective predation or defence, predator confusion, etc. [24]).

However, before the adaptive radiation of the CE, animals in these groups displayed only simple, inflexible bodily movements. Most importantly, the timescale of sensing and action was slow, as it is in extant adult sponges [31]. Though animals of this period could probably sense and react to environmental changes and possibly control their own movement to some degree (as in sponges today [32]), their movement was slow and their behaviours consisted of direct responses to their environment.

### (b) Step 2: The rise of social uncertainty

The new, more complex mechanisms that evolved early in the Cambrian era led to an increase in the range of possible behaviours, creating selective pressures for more processing of information—for cognition. For example, motile predators must be able to subtract their own motion from incoming sensory information to accurately track prey or conspecifics, via a process known as reafference, which may have been one of the earliest functions of neuronal systems [15,33,34]. The interposition of neurons between sensory and motor systems, and a corresponding temporal gap between sensation and action, mark the origin of cognition.

With the emergence of cognitive processes, organisms began making decisions and incorporating more information into decision making, including elements retrieved from early forms of memory. Behaviour was no longer predictable or transparent, and coordination became a new challenge. Cognitive processing also allows for responses to be delayed relative to their motivating stimuli, which makes prediction even more difficult. With movement no longer transparently and directly caused by environmental factors, organisms no longer had a simple mechanistic solution to coordinating their behaviours. The rules by which organisms moved could vary from individual to individual and be modified over the course of one’s lifetime.

At this stage, the very possibility of groups was at risk. If each organism failed to attend to the behaviour of their group members and adjust their behaviour accordingly, they could have become lost in a sea teeming with things to move towards and avoid. Individuals could swim off, move against the current, dart after prey or move away from a predator. What had been slow and predictable became chaotic and unpredictable. Groups would have shattered, along with the adaptive benefits of group living. Just at the point when living in groups would have offered benefits of protection from newly evolved predators, individuals no longer had a method for sticking together. Given the immense value of group living, there was strong selective pressure for something to emerge to support continued collective living.

Cognition and behavioural flexibility created the problem, but they also gave animals the tools they needed to adjust to the increasingly flexible behaviours of others, by learning how to predict more complex behaviours. However, for cognition to work to solve this problem, more of an animal’s cognition had to be directed towards the actions of others.

### (c) Step 3: Closing the predictability gap with consciousness

At this point, we propose that phenomenal consciousness becomes socially adaptive. On the question of the first spark of sentience, we remain silent. Instead, our focus is on the environmental problem that feelings could uniquely solve.

Those organisms that experienced negative affect when group members were not around were motivated to approach conspecifics. This provided the incentive for animals to focus their attention on other organisms and their social signals, allowing them to more accurately predict others’ behaviours. This process is visible in extant animals, whose attention focusing skills may develop partly via social mechanisms (e.g. in young humans [35]), and who are hyper-attuned to social cues [36–38].

With its attention focused on its group mates, an organism could detect discrepancies between its predictions of others’ actions and their actual behaviours. These gave rise to negative affect, as mismatched behaviours risked dissolving the group, which motivated error correction mechanisms to keep the group together. Animals may also have gradually learnt to better predict the behaviours of others in their group, improving future predictions, although we make no claim about when this learning emerged. We take this point as the origin of sociality, as distinct from mere group living. Sociality is the way in which behaviourally flexible organisms design their social interactions, be it a gregarious structure in unique species groups, communities of a variety of different organisms as in multi-species bird flocks or remote and occasional interactions between same-species individuals and regular interaction with other species [39]. Sociality emerges from other-oriented cognition.

An alternative means to maintain group cohesion and its attendant anti-predatory benefits is to behave in a way that is more easily predictable by other members of the group, while ideally still being *un*predictable to potential predators. The process we describe above, of increasing attention to and processing of social cues, would also have acted to increase the complexity of behaviours that animals could display without disrupting collective movement. In extant animals, emotional states are often broadcast as signals to conspecifics (e.g. blushing [40]), further enhancing predictability (knowing another’s emotional state is key to predicting their future actions). This could be considered an evolutionary version of the mindshaping argument, according to which simple behavioural coordination leads to shared internal states that facilitate more complex coordination [41].

Behaviour prediction is an essential skill for cognitive organisms, and the capacities involved in behaviour prediction come in a great variety [42]. What all the methods of behaviour prediction require is some cue that might be taken as a cause of the detected behaviour. Importantly, the ability to use affect to learn and to perform error-correction behaviours requires that animals can identify aspects of the environment relative to which the relevant behaviour must be organized, and so it requires both sensory awareness and valenced affect.

There are three possible stories about the initial appearance of sensory awareness and affective sentience in evolutionary history. It is possible that awareness of sensory stimuli—conspecifics, potential prey and predators, elements of the external world such as light or temperature—evolved first, and only later did organisms gain an ability to attach valenced affect to those conscious representations. Alternatively, animals may have first evolved the ability to have valenced sensations about stimuli that they did not consciously perceive, experiencing a kind of free-floating pleasure or pain before later developing the ability to be aware of sensations. Finally, the two could also have evolved concurrently.

Though our theory is focused on the emergence of sentience and its adaptive benefits, not sensory awareness, our story is consistent with all three of these possibilities. In any of these scenarios, once behavioural flexibility arose, the old methods of coordinating behaviours no longer sufficed. A new problem emerged, but with it came new ways of solving problems. Organisms could learn new means of coordinating behaviour, associating social cues with future actions and finding new ways of keeping groups together. First, however, the organisms needed to value sociality and recognize the chaotic social world as a problem. We hypothesize that consciousness originally served the function of directing attention to social partners, making sociality feel good and isolation feel bad.

## 4. The argument from neuroscience

If some forms of consciousness, such as affect or sensory awareness, evolved as early as we are proposing, then they are a feature of relatively simple neural systems, which is all that existed in the early Cambrian. Debates around the neural correlates of consciousness mostly concern more complex forms than we are considering. However, it is conceivable that sentience could be supported by very simple brains.

At its core, sensory awareness involves reprocessing of some sensory information (or re-presenting it to the brain [43]). In other words, awareness is a function of feedback. In addition to processed sensory information being used to drive action-selection, stimuli that we are aware of are reflected back into the brain. Affect provides an evaluative signal for how to respond to the sensory information. It has been suggested that early neural systems had just such a feedback loop, which was used for reafference ([34]; reafference is also present in extant sponges and ctenophores, supporting its early origins [33]). In reafference, motor information is reprocessed as sensory data to allow for the subtraction of one’s own movement from the external sensory stream. It is possible that the neural structures that underlie this feedback loop were duplicated, perhaps via one of the genome duplication events that occurred during the CE [44], and the extra copy was then used as an initial substrate for sensory awareness. In other words, brains did not evolve sentience owing to specific selective pressures for consciousness; consciousness resulted from an exaptation of existing brain structures, retained because of the social benefits that it made possible.

Modern brains retain close connections between the substrates for social cognition and affect. For example, a wide range of vertebrate species [45], from mammals [46] to reptiles [47] and fish [48], rely on an interconnected set of brain regions to process social information (the ‘social behaviour network’), which partly overlaps with and is strongly connected to the mesolimbic reward system, a key region for emotion (the two areas together are sometimes called the ‘social decision-making network’ [49]).

Additionally, the μ-opioid system, closely involved in the regulation of pain, also plays a central role in modulating many social behaviours (reviewed in [50]). Pain-processing areas of the brain can be activated not only by stimuli that are directly (or potentially) physically harmful, but also by social rejection [51] or by observing another individual in pain [52], and the intensity of such empathetic pain correlates with social closeness to the partner (reviewed in [53]). Empathetic pain activates the same areas of the brain involved in the affective experience of physical pain [54], and it can be reduced using painkillers [55]. The oxytocin system is also closely involved in both increasing prosocial behaviour (possibly by increasing the salience of social cues [56]) and stress reduction [57], and it may play a role in social buffering (a reduction of stress resulting from social contact [58]).

## 5. Deep adaptive alignment argument

A third argument supporting the social origins of consciousness hypothesis comes from a development of James’ adaptive alignment argument, which he gave in response to T.H. Huxley’s claim that while bodily changes impact conscious experience, conscious experience never causes changes to bodily states—just as, in his famous metaphor, the steam engine causes the whistle to produce sound, but the sound has no impact on the function of the engine [59]. As Huxley was a fellow evolutionary theorist, James’ arguments appealed to the shared premise that if consciousness made no causal contribution, it could not have evolved through natural selection. James offers three reasons to think that ‘life-essential, phenomenal pleasures and pains’ [60] are adaptive: they are complexly organized; they are linked with beneficial and harmful brain and bodily states; and these patterns are universal across humans. There is no other explanation for these three features, thus conscious experiences of pain and pleasure must be causally efficacious, and have been selected for.

While James offers this as an argument against epiphenomenalism, it is also an argument for a function of consciousness. However, James was focused on causes of pain and pleasure in terms of physical damage to the body—for the sake of convenience, we will refer to these as ‘bodily pains’. This focus on bodily pain is made explicit in a laundry list of pain examples given by Grant Allen, a contemporary of James, which includes: pain when passing gallstones, breaking nails below the quick, having chapped lips, abscesses, ulcers, cancers, eating excess cayenne pepper and destroying the skin and muscles with acid, among many other more or less gruesome experiences (as discussed in [60, p. 1185]). However, in this list, we do not find the death of one’s young child, losing an aged parent who wanders off in the woods, being forsaken by a lover, having a friend commit suicide, being a survivor of an attack that killed your family or any other kind of social pain. That is, what we can call ‘social pains’ are not considered.

James argues that bodily pains are adaptive not just because of a general adaptive alignment, but also by their deep adaptive alignment—that the more serious the damage, the worse the pain. Passing gallstones is more painful than chapped lips, and gallstones are also more life threatening than chapped lips. Of course, the mapping is not perfect, and we now enjoy many things that are harmful to us as well, but the idea is that there is a rough mapping of degrees of pain and degrees of harm.

Starting with James’ idea of this deep adaptive alignment, we can support the social origins of consciousness hypothesis by showing that social pains are more painful than bodily pains, and that social attention is deeply desired and will be sought out even when it increases the risk of bodily pain. If consciousness was selected for because of its role in facilitating group living at a time of upheaval and increasing unpredictability of individual behaviour, we would expect that social ills are the most aversive. Following James’ logic, it would be quite a coincidence if most existing animals have deep preferences for social goods, and sociality offers immense evolutionary benefits, and yet consciousness was not an early adaptation for social cohesion.

While it has been well established that social isolation causes significant psychological and physical damage, perhaps the strongest evidence comes from preference tests that support the claim that social attention is deeply preferred across a wide range of species, and social isolation is among the most aversive experiences.

One piece of evidence for the relative importance of social attention comes from studies of the importance of social contact. Among the earliest were Harry Harlow’s studies of primates in the 1960s, where he deprived infants of maternal care and socially isolated adult individuals. Harlow’s goal was to better understand human mental disorders by intentionally creating disorders in monkeys and chimpanzees. Infant monkeys were separated from their mothers for 6–12 months and provided a choice between two objects, one a mesh wire ‘mother’ that delivered milk, the other that was soft but delivered no food. The infants preferred the soft ‘mother’, speaking to the deep importance of social interaction and touch in primates. Indeed, when infant monkeys were separated into two conditions—one in which they only had access to the wire ‘mother’ and the other in which they only had access to the cloth ‘mother’—infants in the wire ‘mother’ context showed significant psychological deficits [61]. Subsequent research on the importance of touch in mammals has discovered the existence of nerve fibres called C-tactile afferents that are specially attuned to social touch, particularly affiliative touch such as gentle stroking [62,63]. These have been found in all mammals studied, including primates, pigs, rats, mice, guinea pigs, rabbits and cats [64,65]. Social touch has been identified as a significant moral interest for all animals [66].

Social enrichment is widely understood to be necessary for health and well-being in mammals, and there is a growing awareness of its importance to reptiles and amphibians [67,68]. In formal studies, hamsters will push heavy doors to gain social contact [69], rats prefer social contact to chocolate [70], rhesus monkeys will starve themselves rather than giving a conspecific a shock [71] and mice prefer a cagemate over nesting material [72]. A wide variety of animal models have been studied to show that sociality lowers self-administration of drugs such as cocaine, ethanol and opiates [73], and even fruit flies have been found to prefer healthy foods over alcohol after social sexual interactions [74]. The interpretation is further supported by studies of trade-offs between social interaction and physical pain. In one study, trout, but not goldfish, were found to prefer social contact to avoiding a shock; the trout will suffer increasingly strong shocks to approach a social partner [75]. Lack of social housing in rats has been shown to impact research results on drug trials, leading to significant problems with translation to humans and failures of replicability [76]. Of course, more research will be informative, but together these studies provide corroborative evidence supporting the claim that animals have a deep need for social contact and value it highly enough to (temporarily) forgo even basic necessities such as food and shelter.

## 6. Testing the social origins of consciousness hypothesis

Although we obviously cannot examine the behaviours and cognition of early Cambrian animals, which we propose were already conscious, the social origins of consciousness hypothesis makes some predictions about the structure of consciousness that, if our story is correct, may be observable in extant species as well. We identify two types of tests that can help to corroborate or undermine the social origins of consciousness hypothesis. These tests should be conducted on a wide range of animal species, not just the primates so often used in contemporary consciousness research, but also non-mammalian vertebrates and invertebrate species [77].

*The salience of social stimuli test*. If consciousness evolved to support social cohesion, then we should expect that social cues should be disproportionately salient compared with non-social cues. There is some evidence of this in the human literature: for example, in the propensity to focus on the eyes of others [78] and to use their gaze direction to extract social information [79,80]. This suggests that humans are hard-wired to attend specifically to those features of the social environment that are likely to help predict the future actions of others (e.g. faces showing an emotional expression attract more attention [81]), and there is some evidence that reactions to such social cues engage different brain regions from non-social spatial cues [82]. Though this effect is congruent with our story, it has only been tested in a few species, mostly humans and other mammals. According to our story, the origins of this effect are ancient and should be observable in a very wide range of species, including those often considered non-social.

The hypothesis that social cues are inherently better at attracting attention could be tested in several ways. For example, humans (and other animals) should be more attracted to social cues that are predictive of future behaviour than those that are not, and this distinction may be learnt. Masking effects should be stronger when a social stimulus is used as the mask or weaker when the target to be masked is a social cue. Similarly, since salience is well-known to affect learning, learning that relies on social cues should proceed faster or be remembered better than similar tasks that involve non-social cues.

*The overattribution of agency test*. Agency detection is taken to be innate for many animals [83,84]. Our innate focus on social cues may relate to humans’ propensity to anthropomorphize—viewing even non-social things in the environment as if they were social, using our social skills to make predictions about, for example, the movements of objects (such as in the Heider–Simmel animation; [85]). This research is often characterized as leading to an error—the tendency to overattribute human characteristics to non-humans, including faces on buildings and garbage cans, as well as human-like motivations or social relationships to other species. However, if all animals are specially attuned to social stimuli then anthropomorphism may simply be the human version of a more general feature, namely, the overattribution of agency.

Darwin wrote about the overattribution of agency in animals when he discussed his dog’s reaction to an open parasol that was occasionally moving in the wind [86]. Corroborating Darwin’s report, in experimental contexts, dogs appear to attribute animacy to objects that behave in seemingly agentive ways [87]. While there have been a few studies of overattribution of agency in animals, such as a null study of rhesus monkeys watching a Heider-Simmel type animation [88], there is very little evidence either way that animals across species overattribute agency. Research on agency attribution could be conducted in a wide range of species, using particular stimuli calibrated to the species’ ecological niches and sensory systems. Visual stimuli watched on a computer monitor may not be equally salient to all animal species.

In sum, we suggest that a more robust study of sociality in a wide range of animal species—going far beyond the typical monkey subjects of consciousness research—would help to support or undermine the social origins of consciousness hypothesis.

## 7. Conclusion

In this presentation and defence of the social origins of consciousness hypothesis, we have focused on the adaptive function of sentience. Our suggestion is that social cognition, and more specifically the capacity to coordinate with group members after the rise of cognition, was the original function of consciousness, and as such should be expected to be almost ubiquitous in existing conscious animals. One implication is that self-awareness, rather than understanding of others, should be taken as the major cognitive achievement.

Our three arguments for the hypothesis draw on the observation that sociality is evolutionarily ancient and essential to animals. Threats to sociality require solutions, and one of those solutions will be an emotion-driven motivation to be in the presence of social partners. In modern animals, the neurophysiological structures that regulate pain, a basic type of sentience, also work to process social information and modulate social responses. These physiological connections are reflected in the widespread preference for physical pain over social pain, and the robust evidence that social attention modulates physical pain.

We hope to inspire testing of the hypothesis, but also to inspire including a wider range of animal subjects in the science of consciousness. If consciousness emerged to promote sociality, the study of consciousness in social animals that are already widely used in research, such as nematode worms and fruit flies, would be a promising means of making progress on discovering those properties that are necessary for conscious experience. We also offer a new focus in research on consciousness, one that places attention on the types of stimuli used rather than on the sophistication of the subject’s sensory modalities or cognitive capacities. Testing consciousness using social stimuli in a wide range of social species may allow us to uncover its original function and take some tentative steps towards understanding its mechanisms.

## Data Availability

This article has no additional data.
